# A J-Protein OsDjC46 Interacts with ZFP36 to Participate in ABA-Mediated Antioxidant Defense in Rice

**DOI:** 10.3390/antiox11020207

**Published:** 2022-01-22

**Authors:** Xingxiu Huang, Liping Huang, Xixi Zhao, Jing Jia, Gang Zhang, Mengyao Zhang, Mingyi Jiang

**Affiliations:** 1 College of Life Sciences, Nanjing Agricultural University, Nanjing 210095, China; 2013216005@njau.edu.cn (X.H.); liphuang@fosu.edu.cn (L.H.); 2014116014@njau.edu.cn (X.Z.); 2015116015@njau.edu.cn (J.J.); 2016216005@njau.edu.cn (G.Z.); 2016116095@njau.edu.cn (M.Z.); 2 International Research Center for Environmental Membrane Biology, Foshan University, Foshan 528000, China; 3 National Key Laboratory of Crop Genetics and Germplasm Enhancement, Nanjing Agricultural University, Nanjing 210095, China

**Keywords:** OsDjC46, ZFP36, interaction, ABA, antioxidant defense

## Abstract

ZFP36 has been shown to be involved in ABA-induced antioxidant defense and enhance rice tolerance to drought, salt stress and oxidative stress. Using ZFP36 as bait, a yeast two-hybrid system was used to obtain the interacting protein OsDjC46, which belongs to heat shock protein and usually exists in the form of molecular chaperone, was identified. Further Co-IP (co-immunoprecipitation), BiFC (bimolecular fluorescence complement) and GST (glutathione-S-transferase) pull-down experiments verified that ZFP36 interacted with OsDjC46 in vivo and in vitro. Heat shock protein has been shown to increase plant resistance to stresses, but whether OsDjC46 was a key factor in plant response to various stresses has not been reported. Here, various stimuli, such as abscisic acid (ABA), hydrogen peroxidase (H_2_O_2_), polyethylene (PEG) and sodium chloride (NaCl) markedly induced the expression of *OsDjC46* in the seedlings. Overexpression of *OsDjC46* in rice can enhance the tolerance to salinity and drought; in contrast, knockout of *OsDjC46* rice plants was more sensitive to salt stress and drought. Further investigation revealed that OsDjC46 could participate in regulating the expression and activities of antioxidant of SOD and CAT under drought and salt stress. Taken together, these findings reveal a novel function of OsDjC46 in adjusting ABA-induced antioxidant defense.

## 1. Introduction

Drought and salt stress in nature’s ecosystems seriously affect the yield and quality of crops. Abscisic acid, a plant hormone, plays an important role in plant response to various abiotic stresses (drought, high salt, cold injury, etc.). Plant tolerance to various stresses is a process of very complex signal transduction, and a large number of corresponding genes are up- or down-expressed. Additionally, some key transcription factors are involved [1,2,3,4,5]. Previously, ZFP36, a zinc finger protein transcription factor, was shown to be involved in ABA-induced antioxidant defense and play a positive regulatory role in drought and salt stress in rice [6].

Many studies have shown that transcriptional regulation is one of the most important ways for plants to respond to and adapt to stress. Some transcription factors, such as WRKY, AP2/ERF, NAC, ZFP and MYB, have been shown to be involved in ABA and water stress responses [7,8,9,10]. Transcription factors are mainly responsible for transcriptional activation or inhibition of ABA and stress signal transduction and control the expression of downstream genes. ZFPs are a large family, and there are 189 members in rice, which have been shown to play an important role in plant responses to oxidative and abiotic stresses. Studies have shown that C2H2 ZFPs plays a crucial role in crop growth and development, hormone response and stress response [11,12,13]. It has been proved that ZFP36 [14], ZFP179 [15], ZFP182 [16,17], ZFP245 [16,18,19], ZFP213 [14] and OsDRZ1 [20] participate in the response of rice to salinity, drought and oxidative stress and regulate reactive oxygen species (ROS) signal transduction. ZFP245 was the first identified C2H2-type zinc finger protein in rice which was induced by cold and drought stresses [21], while ZFP182 is a salt-inducible zinc finger gene that can improve salt tolerance in transgenic tobacco and rice plants [22]. ZFP182 and ZFP36 have been proved to be ABA- and H_2_O_2_-responsive genes that participate in ABA-induced antioxidant defense. Meanwhile, H_2_O_2_, MAPK and NADPH oxidase involved in ABA signal transduction are regulated by ZFP36, but the specific mechanism of action is still obscure. In this study, we performed a yeast two-hybrid assay and many proteins interacting with ZFP36 were found, OsDjC46, a heat shock protein, was further identified to be the potential interacting protein of ZFP36 to participate in ABA-related response.

Due to its unique DnaJ structure, OsDjC46, also known as DnaJ protein, belongs to the chaperone protein of the Hsp70 family [23,24] and is widely involved in various cellular processes [25,26]. A recent genomic study revealed that there are 115 putative J-proteins containing nine clades in rice. Various DnaJ are regulated under different stress conditions, and the genes are expressed constitutively or regulated developmentally. As a kind of helper protein, J-proteins usually works as a molecular chaperone [27]. It has been reported that the DnaJ protein is involved in abiotic and biological stress response and is a critical chaperone protein in plant growth, development and response to various environment stresses [28,29,30,31]. As a protein companion, J-protein can act independently and is involved in the REDOX reaction process [32]. AtDjA3 in Arabidopsis is involved in seed development and abiotic stress tolerance [33]. J-proteins form a large family in rice; however, little has been reported on this gene family in rice.

OsDjC46 protein belongs to a J-protein of the third category (Hsp40 family); the sequence homology of OsDjC46(LOC_Os04g59060) has been shown in previous studies [34]. By analyzing (https://www.ncbi.nlm.nih.gov/Structure/cdd/wrpsb.cgi, accessed on 10 October 2017), it has been found that the J-domain of OsDjC46 is located at the *C*-terminal, which is in accordance with type C. Our study also shows that OsDjC46 is involved in the regulation of antioxidant enzyme activity related to drought and salt stress by protecting the cell membrane damage from the damage caused by peroxidation of plasma membrane and by maintaining the balance of osmotic pressure of cells and removing reactive oxygen species, thus improving plants’ tolerance to adverse environments.

## 2. Materials and Methods

### 2.1. Plant Materials and Treatments

Full and consistent rice seeds (*Oryza sativa L. sub. japonica cv. Nipponbare*) were selected and then were grown hydroponically with a culture solution in the illumination incubator with the temperature of 28 °C (day) to 22 °C (night) (RH90%, day/night for 16 h/8 h). After the leaves were fully expanded (the second leaves), using 100 μmol·L^−1^ ABA (Sigma-Aldrich, St. Louis, MO, USA), 10 mmol·L^−1^ H_2_O_2_ or 10 mM CaCl_2_, 150 mM NaCl and 20% PEG 6000 to treat the plants for the indicated time. To obtain the effects of inhibitors, the rice plants were pre-treated with DPI (100 μmol·L^−1^) (Sigma-Aldrich), CAT (200 U/mL) (Takara Bio, Beijing, China), DMTU (10 mmol·L^−1^) (Sigma-Aldrich), EGTA (5 mM) (Sigma-Aldrich), TFP (100 μΜ) (ALEXIS Biochemicals, Lawson, Switzerland), W7 (200 μΜ) (ALEXIS Biochemicals), W5 (200 μΜ) (ALEXIS Biochemicals), KN-92 (1 μΜ) (ALEXIS Biochemicals) and KN-93 (1 μΜ) (ALEXIS Biochemicals) for 4 h and then treated with 100 μmol·L^−1^ ABA under the same conditions as described above. After treatments, the rice leaves were collected, and liquid N_2_ was used to frozen the materials immediately for further analysis.

### 2.2. RNA Extraction and RT-qPCR Analysis

Plant tissues preserved in the liquid N_2_ were ground into powder; then, the RNAiso (Takara Bio) was used to extract the total RNA, and a reverse transcript kit (Takara Bio) was used to obtain cDNA according to the manufacturer’s instructions. RT-qPCR reactions was performed using the SYBR Premix Ex TaqTM (Takara Bio) according to the manufacturer’s protocol. Additionally, the expression was calculated by using the values of 2^−ΔΔCT^.

### 2.3. Subcellular Localization

The cDNA fragment of *OsDjC46* was cloned from the rice cDNA library with a pair of specific primers, and the 35S:OsDjC46-YFP was constructed as the instantaneous expression vector to detect the cell localization of OsDjC46 in the cells of rice protoplast. Purified 35S: Flag-OsDjC46-GFP vector by lithium chloride, the vector was bombarded into onion epidermis cells by a gene gun [35]. OsDjC46-1300 (GFP) fusion construct was transiently introduced into *Nicotiana benthamiana* leaves (25-day-old) by Agrobacterium tumefaciens strain EHA105-mediated transformation [36]. All the fluorescence was observed by laser confocal microscope.

### 2.4. Verifying the Interaction between OsDjC46 and ZFP36

For the yeast two-hybrid assay, the Clontech Y2H Gold yeast two-hybrid system was used according to the manufacturer’s protocol (Clontech, Shiga, Japan). Y2H Gold (Saccharomyces cerevisiae strain) transformed with the indicated construct (bait) was grown on the SD/-Trp, and the Y187 transformed with indicated constructs (prey) was grown on the synthetic dropout media SD/-Leu. Then, the two transformed yeast strains were mated with 2 × YPDA and were grown on the synthetic dropout SD/-Trp-Leu (SD/-T-L) at the temperature of 30 °C for 3–5 day. Then, to test the growth condition and blue colony of the transformants by transferring to the synthetic dropout media SD/-Trp-Leu-His-Ade/X-a-gal/AbA^r^.

For BiFC assay, ZFP36 construct (ZFP36-YFPC) without stop codon was cloned into the BamHI-KpnI sites of pSPYCE to generate ZFP36-YFPC plasmid, and the coding sequence of OsDjC46 without stop codon was amplified and cloned at the KpnI-BamHI sites in pSPYNE to form OsDjC46-YFPN construct. OsDjC46-YFPN combined with ZFP36-YFPC was bombarded into onion (*Allium cepa* L.) epidermal layer cells, and the yellow fluorescence signal was captured by a ZEN 2012 Zeiss laser confocal microscope.

For Co-IP assay, OsDjC46-Flag-1300 and ZFP36-His-1300 were constructed (35S: Flag- OsDjC46-GFP or 35S: His-ZFP36), respectively, and were transformed into the Agrobacterium tumefaciens strain GV3101. Then, they were transiently transformed separately or co-infiltrated into the tobacco leaves (about 28-day-old). After infection for 3 days, the infected tobacco leaves were collected, and then the total proteins were extracted. The anti-Flag antibody and protein A magnetic beads (Promega, Madison, WI, USA) were added to incubate for 3 h and collected the sample with the buffer (1 × SDS), and then the Western blot test was performed.

For GST pull-down assay, the fusion proteins of GST or GST-OsDjC46 fusion proteins were immobilized on GST beads (Promega) and then using His-tagged ZFP36 (constructed by pET28a-ZFP36) binding buffer to incubate the two proteins. the GST beads were washed with wash buffer several times, using 15% SDS-PAGE to elute and separate the binding proteins, and then immunoblotting was employed with anti-His antibody to identify the bands. 

### 2.5. The Generation of Transgenic Plants

OsDjC46 knockout rice was constructed with CRISPR/Cas9 technology [37,38]. The CRISPR/Cas9 binary expression vector was constructed by using the pRGEB31 plasmid as vector. Agrobacterium-mediated genetic materials were constructed. Full-length OsDjC46 sequence and its exon sequence were searched in the rice library and were compared via BLAST of NCBI. The OsDjC46 gene site was mapped, and some common enzyme-cutting sites in OsDjC46 were searched in software of Vector NT1. LOC_Os04g59060 (OsDjC46 gene number in rice) was submitted in CRISPR-PLANT (https://www.genome.arizona.edu/crispr/CRISPRsearch.html, accessed on 9 September 2016) to find all gRNA (grade “class0.0/1.0”) [39]. Then, the suitable gRNA was selected for cloning, and the successfully sequenced plasmid was transformed into the Agrobacterium tumefaciens strain EHA105 [36]. It was verified that the knockout material could be compared with the original sgRNA fragment by PCR product delivery sequencing method, so as to determine whether the material had mutated. The identification result is presented in Figure S2.

The full-length of OsDjC46 fused into the pSUPER1300 vector using the restriction enzyme sites of HindIII and KpnI (Takara Bio) to construct OsDjC46-overexpression vector was also transformed into A. tumefaciens EHA105. The OsDjC46 overexpressed material was observed with green fluorescence in plant cells under laser microscope to detect whether the material had been successfully obtained.

### 2.6. Enzyme Assays

Rice protoplasts were homogenized in 50 mM and pH 7.0 potassium phosphate buffer containing 1 mM EDTA and 1% polyvinylpyrrolidone. The homogenate was centrifuged at 12,000× *g* (4 °C) for 30 min, and the supernatant was immediately used for the antioxidant enzyme tests. The total activities of superoxide dismutase (SOD) and catalase (CAT) were determined as described previously [40].

### 2.7. Determination of Cell Membrane Oxidative Damage

The content of malondialdehyde can be determined using cell membrane oxidation damage. The rice leaves were treated with PEG or NaCl and using 10% trichloroacetic acid to homogenize, as described by Hodges [41]. The electrolyte leakage can be measured as reported previously by Jiang and Zhang [40], and the rate of ion leakage was calculated as the percentage of initial conductivity divided by the total conductivity.

### 2.8. Drought and Salt Tolerance Assays

T3 transgenic materials were selected, and then hydroponics were chosen for cultivation. A 150 mM measure of NaCl and 20% PEG6000 were used to simulate salt and drought stress. Two weeks later, the rice seedlings were treated in stressed condition for 10 day, after cleaning with clean water and culture in nutrient solution for 15 day. Rice growth and performance were monitored, including survival rate, plant height, fresh weight and root length.

### 2.9. Protoplast Isolation

The rice plants were grown in a dark environment at 28 °C for 2 weeks. When plants were ~4–8 inches tall, the protoplast from leaf and tissue were isolated. Washing solution was added to clean and filter the enzyme solution into a centrifugal tube with filter cloth and then centrifuged at room temperature to collect protoplasts. The protoplast separation method was described by Zhang [17].

## 3. Results

### 3.1. Identification of OsDjC46 as an Interacting Protein of ZFP36

**Figure 1:**
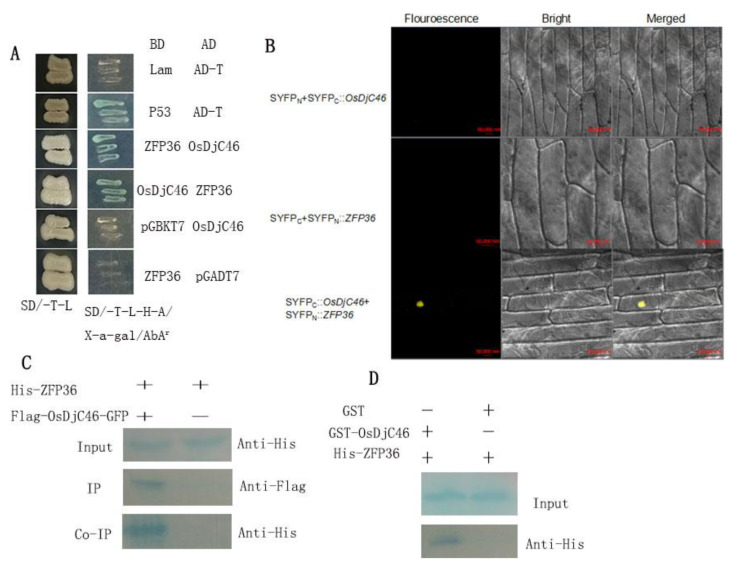
The interaction between OsDjC46 and ZFP36 in vivo and in vitro. (**A**) Yeast two-hybrid test. The positive control was BD-P53/AD-T, while BD-Lam/AD-T was used as the negative control. SD/-Leu-Trp medium was used to grow the transformed yeast, and SD-Leu-Trp-His-Ade/X-a-gal/AbA^r^ was used as the selective medium. AD and BD are the activating and binding regions of transcription factors, respectively; SD, synthetic dropout media. (**B**) BiFC test of OsDjC46 and ZFP36 interaction in onion (*Allium cepa* L.) epidermal layer cells. A confocal laser scanning microscope was used to detect the yellow fluorescence protein (YFP) signals. Scale bars = 50 µm. (**C**) Co-IP assay. Twenty-five-day-old tobacco epidermal layer cells were transformed with 35S: His-ZFP36 alone, or 35S: Flag- OsDjC46-GFP alone or 35S: Flag- OsDjC46-GFP and 35S: His-ZFP36 simultaneously. Anti-Flag was used to immunoprecipitate the CP (crude protein) and the Western blot with anti-His antibody was used to check the proteins. (**D**) Pull-down assay. GST affinity Magnetic™ beads make the GST alone or GST- OsDjC46 fusion protein bind to the pET28a-His-ZFP36. Additionally, then used the Western blot with anti-His antibody to detect the result.

To isolate interacting proteins of ZFP36 in rice (*Oryza sativa L. sub. japonica cv. Nipponbare*), the full length of ZFP36 expressed as a fusion protein with the GAL4 DNA-binding domain (GAL4-BD) was used as a bait to perform the yeast two-hybrid (Y2H) screen of the rice leaf cDNA library fused to the GAL4 activation domain (GAL4-AD). Approximately 106 yeast transformants were screened, and 5 of 59 positive clones were identified to contain the same cDNA with its full-length sequence encoding a protein of 275 amino acids designated as OsDjC46 (LOC_Os04g59060). The interaction of OsDjC46 with ZFP36 in the additional yeast two-hybrid assay is shown in Figure 1A. As shown in Figure S1, we showed that ZFP36 interacts with OsDjC46 *N*-terminal domain but not *C*-terminal domain. The interaction shows that the J-domain functions as a heat shock protein [30,31,32,33,34], or it may bind to other proteins when exposed, but the specific mechanism of action is still obscure.

In order to further ensure the authenticity of the interaction between OsDjC46 and ZFP36, both in vitro and in vivo assays were carried out. BiFC experiments was used to verify the interaction in onion (*Allium cepa* L.) epidermal layer cells. The pSPYCE vector was recombined to obtain SYFPN::OsDjC46 vector and SYFPC::ZFP36. The fluorescence was observed when SYFPN::OsDjC46 and SYFPC::ZFP36 were co-expressed (Figure 1B). However, co-expression of SYFPN and SYFPC::ZFP36 or SYFPN::OsDjC46 and SYFPC did not observe the fluorescence. Thus, the BiFC assay revealed that OsDjC46 interacted with ZFP36, and the interaction occurred in the nucleus (Figure 1B).

Co-immunoprecipitation (Co-IP) test was used to confirm the interaction between OsDjC46 and ZFP36 in *Nicotiana benthamiana* leaves. Flag-OsDjC46-GFP and ZFP36-His were co-expressed in tobacco leaves, and ZFP36-His were expressed alone separately. CP (crude protein) extracts of *Nicotiana tabacum* leaves were immunoprecipitated by anti-Flag (or anti-His) antibody, and then immunoblotting was performed with anti-His (or anti-Flag) antibody. As revealed in Figure 1C, we could detect the ZFP36-His, further confirming the interaction between the two proteins of OsDjC46 and ZFP36 in vivo.

For in vitro assay, a GST pull-down test was used. In *Escherichia coli*, the recombinant OsDjC46 and GST (glutathione S-transferase) fusion protein and ZFP36 tagged with poly-His were produced, then purified with Magnetic™ GST or His affinity Magnetic™ beads. The results showed that His-ZFP36 was not captured by GST beads, while His-ZFP36 was bound to beads with GST-OsDjC46, indicating that OsDjC46 and ZFP36 could interact in vitro (Figure 1D).

These results collectively reveal that OsDjC46 interacted with ZFP36 in vivo and in vitro.

### 3.2. OsDjC46 Characterization

**Figure 2:**
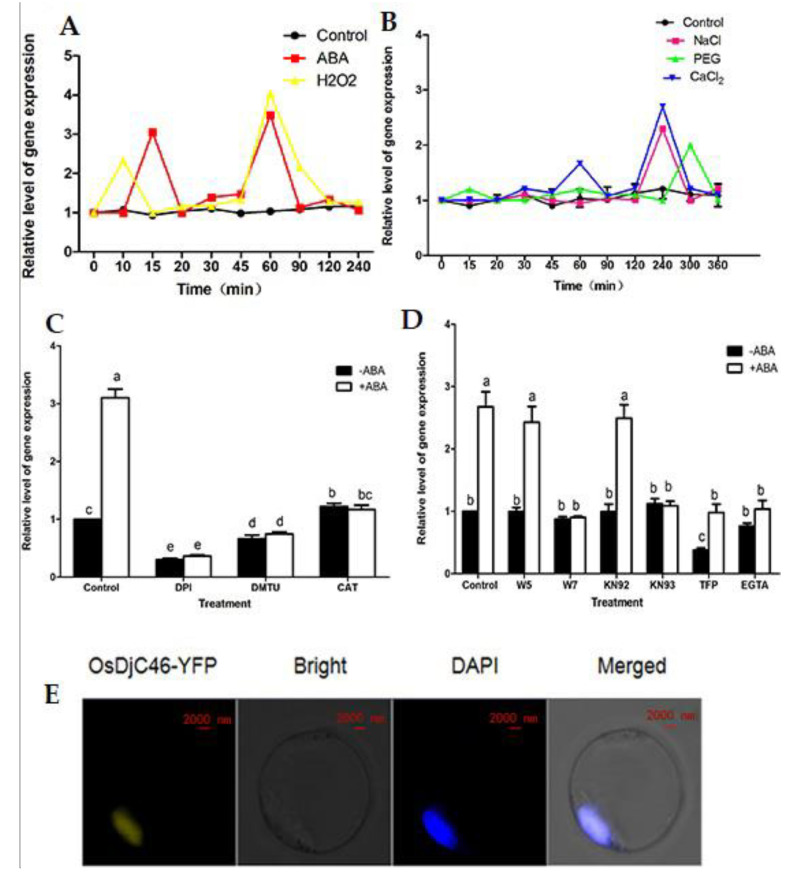
The subcellular localization and expression pattern of OsDjC46. (**A,B**) The expression of OsDjC46 under various stimuli, CaCl_2_, ABA, H_2_O_2_, NaCl and PEG stresses. RT-qPCR was used to measure the results with the special primers listed in Supplementary Table S1, and *OsActin* was used as the reference control. (**C**,**D**) The expression of OsDjC46 with different inhibitors. DPI, DMTU and CAT were scavengers and inhibitors of H_2_O_2_. EGTA was a calcium channel blocker, while TFP and W7 were CaM antagonists, W5 was a negative control of W7, KN-93 was a CCaMK (calcium and Ca^2+^/calmodulin-dependent protein kinase) inhibitor and KN92 was a negative control of KN-93. (**E**) The subcellular localization of OsDjC46 in rice protoplast cells. 35S:YFP as the control was transiently transformed via PEG-calcium method. Using the confocal laser scanning microscope to detect the yellow fluorescence protein (YFP). Scale bars = 2 mm. Values are means ± SD of three independent assays. At the level of *p* < 0.05, there was no significant difference between the same letter according to Duncan’s multiple comparisons.

In order to further understand the function of OsDjC46 in rice for the antioxidant defense, the basic biological information of OsDjC46 was investigated. Rice seedlings were treated with ABA, H_2_O_2_, Ca^2+^, PEG and NaCl, and the expression of OsDjC46 in the leaves was detected. According to the results of RT-qPCR analysis, the expression of OsDjC46 was induced within 4–5 h under drought or salt stress (Figure 2B).

After ABA treatment, the expression of OsDjC46 increased rapidly at 15 min and 60 min compared with the control. In this study, we found that *OsDjC46* has a similar expression pattern with *ZFP36*, its interacting protein, in which the expression of ZFP36 also has two peaks [6]. We think there may be a cross-talk between OsDjC46, ZFP36, ABA and H_2_O_2_. On the one hand, ZFP36 promotes ABA biosynthesis and induces H_2_O_2_ accumulation. On the other hand, the expression of *OsDjC46* is positive feedback-regulated by ABA and H_2_O_2_. Therefore, the expression of *OsDjC46* has two peaks. Under the treatment of H_2_O_2_, OsDjC46 expression peaked at 10 min, then began to be down-regulated and reached the maximum peak at 60 min, then gradually declined. This up-regulation change was rapid and instantaneous, and the peak time in the first stage was earlier than that after ABA treatment, suggesting that might be related to H_2_O_2_ (Figure 2A). These data suggest that OsDjC46 may be involved in the ABA signaling pathway (Figure 2A).

To further survey the mechanism of OsDjC46 involved in ABA signaling pathway, rice plants were pre-treated with the H_2_O_2_ scavengers (DMTU, CAT) and inhibitors (DPI), which could effectively clear H_2_O_2_ before ABA treatment. As shown in Figure 2B, the expression of OsDjC46 was no longer increased with exogenous ABA. This result implied that the expression of OsDjC46 could be further induced by ABA because of the endogenous H_2_O_2_ (Figure 2C).

Ca^2+^ is an important component of ABA signal transduction system, which plays a pivotal role in regulating the signal transduction of reactive oxygen species (ROS) (Zhu et al. 2016). Under CaCl_2_ treatment, OsDjC46 expression reached its maximum peak at 180 min (Figure 2A). However, the pretreatment of rice seedlings with calcium-related blockers, antagonists or inhibitors (W7, KN93, TFP, EGTA) had no significant effect on the expression of OsDjC46, while the treatment with ABA showed no significant increase in OsDjC46 expression compared with the control group (Figure 2D). These calcium-related blockers, antagonists or inhibitors can inhibit the expression of OsDjC46 induced by ABA. Taken together, the expression of OsDjC46 induced by ABA was possibly mediated by impeding Ca^2+^ signaling in the signal transduction of ROS.

To investigate the subcellular localization of OsDjC46, the recombinant vector of 35S:OsDjC46-YFP was transiently expressed in rice protoplast cells by the PEG-calcium method as described. The yellow fluorescence in protoplast was observed by laser confocal microscope, and the nuclear localization of OsDjC46-YFP was verified by its co-localization with the nuclear dye DAPI (Figure 2E).

### 3.3. The Physiological and Biochemical Indexes and Phenotypic Analysis of Transgenic Plants under Drought and Salt Stress

**Figure 3:**
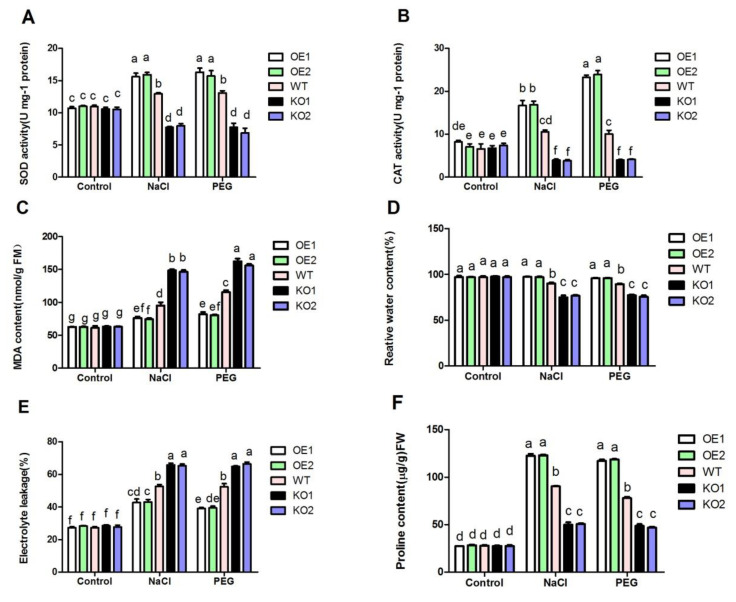
*OsDjC46*-transgenic plants respond to drought and salt stress. SOD (**A**) and CAT (**B**) activity. This is ubiquitous SOD or CAT activity, but not specific activity. Because SOD and CAT are essential for protection against drought and salt stress, we examined SOD and CAT activities in rice. MDA content (**C**), the percentage of electrolyte leakage (**E**), relative water content (**D**) and proline content (**F**) were the indexes of cell membrane damage. Twenty percent PEG 6000 was used as drought stress and 150 mM NaCl as salt stress. Two-week-old rice seedlings were treated under drought and salt stress for 2 days, and then the leaves were collected for the assays of cell membrane damage indexes. The data are the average of three independent experiments. At the level of *p* < 0.05, there was no significant difference between the same letters according to Duncan’s multiple comparisons.

Under drought and salt stress, the expression and activities of SOD and CAT of transgenic plants were measured. As shown in Figure 3A,B, the activities of SOD and CAT in *OsDjC46* knockout plants were lower than those of wild-type (WT) plants, while the activities of SOD and CAT in OsDjC46-overexpression plants were higher than that in WT plants. SOD and CAT were two important enzymes in the process of antioxidant defense, which further indicated that OsDjC46 was involved in regulating the activities of antioxidant defense enzyme under drought and salt stress.

Adverse environments lead to excessive accumulation of free radicals in cells and result in membrane lipid peroxidation, and destroying the balance of intracellular environment. MDA is a product of peroxidation of plasma membrane after a cell membrane is damaged. Its content reflects the extent of cell membrane damage, while the electrical conductivity of leaves can also reflect the damage of cell membrane and its change in permeability. As shown in Figure 3C, NaCl and PEG treatment could up-regulate the antioxidant defense system of plants and increase the damage of plasma membrane. Compared with WT plants, the mutant had higher content of MDA (Figure 3D) and electrical conductivity (Figure 3E), indicating that the plasma membrane was damaged more seriously. However, *OsDjC46* overexpressed plants had lower content of MDA, indicating that overexpressing *OsDjC46* in rice could enhance the activity of antioxidant defense and result in less damage to the plasma membrane from drought and salt stress, while knockout of *OsDjC46* in rice could decrease the activity of antioxidant defense, resulting in more damage to the plasma membrane.

Under stressed environments, especially drought and salt stress, leaves will curl and lose water. Therefore, relative water content (RWC) is a good indicator to reflect plants’ tolerance to stresses. As shown in Figure 3D, under normal conditions, there was no significant difference between *OsDjC46*-overexpressed and -knockout plants compared with WT plants. However, after NaCl and PEG treatment, the RWC of OsDjC46-overexpressed plants was higher than that of WT plants, while the mutant had lower content of RWC compared with WT plants.

Drought and salt stress can change the osmotic pressure of the surrounding environment of cells, so that it can affect the growth and development of plants. Proline in plants can increase the concentration of cytoplasm, change the osmotic pressure and reduce the damage of osmotic stress to plant cells. Proline accumulation under osmotic stress can not only maintain osmotic balance and membrane structure stability but also plays a role in regulating REDOX reaction or scavenging reactive oxygen species [42]. At the same time, proline can act as a scavenger of reactive oxygen species. As shown in Figure 3F, under drought and salt stress, the proline content in OsDjC46 mutant was significantly lower, while the content in *OsDjC46*-overexpressed plants was higher than that in WT plants. These results indicated that OsDjC46 participated in modulating the proline accumulation in plants to maintain osmotic pressure balance and remove reactive oxygen species under stress, so as to improve plant tolerance. The results showed that OsDjC46 was involved in the response of rice to drought and salt stress by regulating antioxidant defense system and the content of RWC, proline and the electrolyte leakage rate. When the temperature was 25 °C, the osmotic potential of 20% PEG6000 was −0.65 MPa, and that of 150 mM NaCl was −0.78 MPa. PEG and NaCl treatments both cause osmotic stress, and sodium chloride also has ionic toxicity. Osmotic stress causes water loss in plants, resulting in changes in SOD, CAT activity, MDA content, electrolyte leakage, etc.

**Figure 4:**
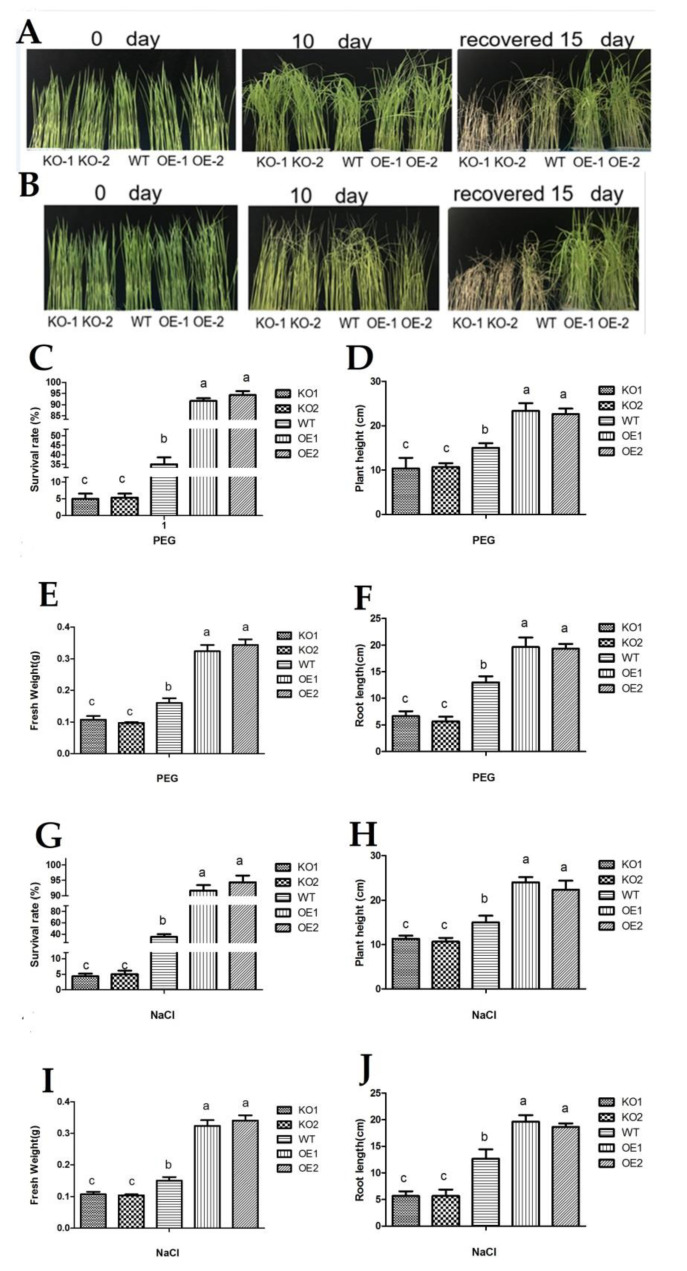
Phenotypic analysis of OsDjC46-transgenic plants under drought and salt stress. (**A**) Photographs of plants grown under drought stress and (**B**) salt stress. Two-week-old rice seedlings were treated with 150 mM NaCl and 20% PEG 6000 for 10 days and then left to recover for 15 days. (**C**–**J**) Experiments were repeated for three times, the fresh weight, plant height and root length of the material were statistically analyzed. Values are means ± SD of three independent assays. At the level of *p* < 0.05, there was no significant difference between the same letter according to Duncan’s multiple comparisons.

20% PEG6000 was used to simulate drought stress. When the plants were at the two-leaf stage, they were exposed to 20% PEG6000 or 150 mM NaCl (Figure 4A,B). Phenotypic analysis revealed that leaves were yellow and curly after treatment for 10 days; however, OsDjC46 mutant had a lower survival rate (Figure 4C,G) than WT plants, while OsDjC46-overexpressed plants had higher survival rate compared with WT plants after rehydration for 15 days. At the same time, the fresh weight (Figure 4E,I), plant height (Figure 4D,H) and root length (Figure 4F,J) of OsDjC46-overexpressed plants were higher than in WT plants, while the OsDjC46 mutant had a lower fresh weight (Figure 4E,I), plant height (Figure 4D,H) and root length (Figure 4F,J) than WT plants. In our study, PEG or NaCl was used to simulate drought or salt stress, respectively. Although these two stresses show the same pattern of effects, this may be due to the same or different mechanisms. Both PEG and NaCl treatments caused osmotic stress; however, NaCl treatments also showed ionic toxicity.

## 4. Discussion

Plants are sessile and usually exposed to various hostile environments, such as drought, high temperature and salt. Plants have evolved complicated mechanism to cope with these adversity environments.

Transcription factors (TFs) play a key role in the response of plants to various stresses. One of the most important TFs is zinc finger protein (ZFPs) [11,12,13]. Plenty of studies showed that ZFPs play a vital role in modulating plants response to various stresses [6,15,16,17,18,19,20]. There are 189 ZFPs in rice [12], and one of the most studied ones is ZFP36. ZFP36 has been proved to be ABA- and H_2_O_2_-responsive genes that participate in ABA-induced antioxidant defense [6], broad-spectrum blast resistance [5], seed germination [43] and the interaction of the late embryogenesis protein involved in drought and salt stress [44]. Since ZFP36 plays a vital role in plant growth, the issue of whether there are other interacting proteins of ZFP36 deserves further investigation. Here, ZFP36 was used as a bait to perform yeast two-hybrid assay screening of the rice cDNA library to find the interacting proteins, and OsDjC46, which belongs to DnaJ proteins, was explored.

Many studies have revealed that DnaJ proteins are involved in various stresses. DnaJ codes a kind of heat shock protein (Hsp) trigged by heat, and in the context of other stresses, Hsps accumulate in the organism [45,46] and regulate the heat tolerance of plants [47]. There are six groups of Hsps—Hsp100, Hsp90, Hsp70, Hsp60, Hsp40/J-protein and small Hsp (sHsp/Hsp20)—based on their molecular weight [23,46]. Interestingly, Hsp40/J-proteins have been often regarded as the obligate partners of Hsp70s, because if there is are Hsp70s, J-proteins cannot work well, and if there are no J-proteins, Hsp70 cannot work well either; thus, J-proteins are the co-chaperones of Hsp70, and the activity of Hsp70 is regulated by J-proteins [48,49]. In this study, we also found that the expression of *OsDjC46* was also induced by ABA and H_2_O_2_ (Figure 2A), as was ZFP36. Previous work demonstrated that ZFP36 was involved in ABA-induced antioxidant defense. So is there any possibility that OsDjC46 is also a factor in ABA-induced antioxidant defense? First, the interaction between ZFP36 and OsDjC46 was further verified by in vivo and in vitro, and the authenticity of the interaction was confirmed, as shown in Figure 1. Additionally, subcellular localization assay showed that OsDjC46 was located in the nucleus of rice, just like ZFP36, and could reconfirm the interaction between OsDjC46 and ZFP36, fully or partially. Then, PEG and NaCl were used for to simulate drought and salt stress. We also found that the expression of OsDjC46 was induced by drought and salt stress, which were simulated by 20% PEG6000 and 150 mM NaCl. The results from using H_2_O_2_-related inhibitors and scavenging agents showed that OsDjC46 was involved in ABA-induced generation of H_2_O_2_ [50,51,52]. Ca^2+^, as an important component of the ABA signal transduction system, could regulate the signal transduction of reactive oxygen species (ROS). Our further investigation revealed that OsDjC46 expression was up-regulated by Ca^2+^ [53,54,55]. Additionally, the results of pretreatment of rice seedlings with different Ca^2+^-related inhibitors followed by ABA treatment indicated that OsDjC46 induced by Ca^2+^ were partially mediated by ABA defense (Figure 2B).

## 5. Conclusions

According to the phenotypic analysis and physiological index data of overexpressed *OsDjC46* and *OsDjC46* mutants under drought and salt stress, we could conclude that OsDjC46 participates in the response to drought and salt stress, partly relying on ABA-mediated antioxidant defense via interacting with ZFP36.

## Data Availability

Data is contained within the article.

## Supplementary Materials

The following supporting information can be downloaded at: https://www.mdpi.com/article/10.3390/antiox11020207/s1, Figure S1: Yeast two-hybrid test. The positive control was BD-P53/AD-T while BD-Lam/AD-T was used as the negative control. SD-Leu-Trp-His-Ade/X-a-gal/AbAr was used as the selective medium. AD and BD are the activating and binding regions of transcription factors, respectively; SD, synthetic dropout media. The colonies of ZFP36-AD and OsDJC46-N-BD were blue as those of the positive control in the four-deficiency medium. OsDJC46-N was connected to pGBKT7 and fused with ZFP36-AD. There was an interaction between ZFP36-AD and OsDJC46-N. ZFP36-AD could activate MEL1 in BD and make its fused colony turn blue in the tetra-deficient medium with X-α-Gal/AbA^r^. However, after fusion and fusion of OsDJC46-C-BD and ZFP36-AD, yeast colonies did not show blue, indicating that OsDJC46-C-BD and ZFP36-AD did not interact. Figure S2: (A) is the result of mutant material identification, and (B) is the result of overexpression material identification. Table S1: Primers in this study.
