# Supplementary material for: A J-Protein OsDjC46 Interacts with ZFP36 to Participate in ABA-Mediated Antioxidant Defense in Rice
Source: Antioxidants (Basel). 2022 Jan 22;11(2):207. doi: 10.3390/antiox11020207 (PMC8868554; doi:10.3390/antiox11020207)
Supplement: Supplementary file 1 [file antioxidants-11-00207-s001.zip › antioxidants-1511080-supplementary.pdf]

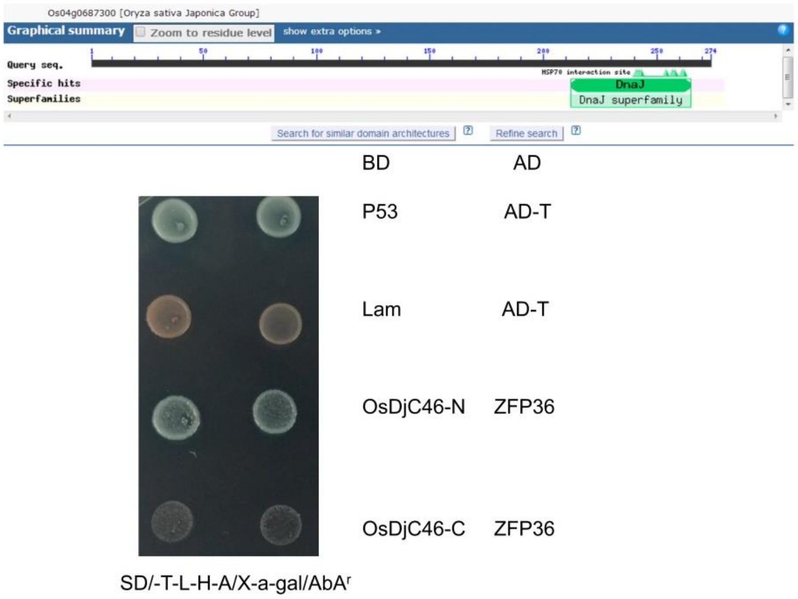

Figure S1. Yeast two-hybrid test.The positive control was BD-P53/AD-T while BD-Lam/AD-T was used as the negative control. SD-Leu-Trp-His-Ade/X-a-gal/AbAr was used as the selective medium. AD and BD are the activating and binding regions of transcription factors respectively; SD, synthetic dropout media. The colonies of ZFP36-AD and OsDJC46-N-BD were blue as those of the positive control in the four-deficiency medium. OsDJC46-N was connected to pGBKT7 and fused with ZFP36-AD. There was an interaction between ZFP36-AD and OsDJC46-N. ZFP36-AD could activate MEL1 in BD and make its fused colony turn blue in the tetra-deficient medium with X-α-Gal/AbA^{r}. However, after fusion and fusion of OsDJC46-C-BD and ZFP36-AD, yeast colonies did not show blue, indicating that OsDJC46-C-BD and ZFP36-AD did not interact.

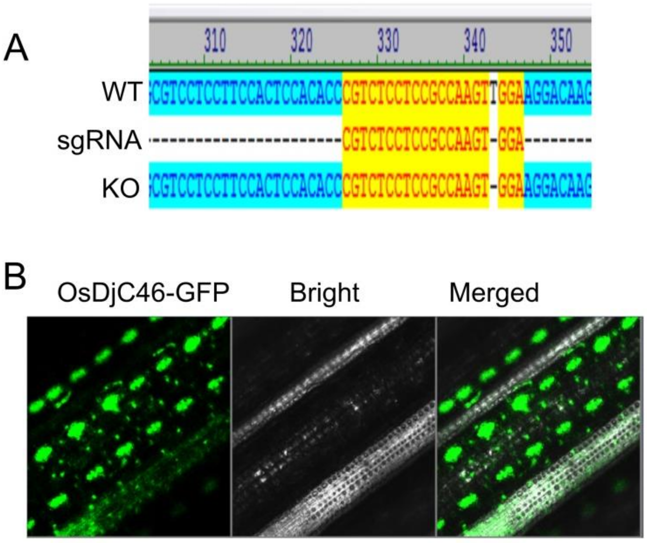

Figure S2. (**A**) is the result of mutant material identification, (**B**) is the result of overexpression material identification.

Table S1. Primers in this study.

**Abbreviation**	**Sequence（5′-3′）**	**Description**
OsDjC46	TCTTCCGTGAGGAGTATTATGTG	Forward
OsDjC46	TTCATCGTCACTATCGCTACAA	Reverse
Actin	CGACCACCTTGATCTTCATGCTGCTA	Forward
Actin	CTTCATAGGAATGGAAGCTGCGGGTA	Reverse
